# Reply to ‘A reexamination on how behavioral interventions can promote household action to limit climate change'

**DOI:** 10.1038/s41467-020-14650-0

**Published:** 2020-02-14

**Authors:** Claudia F. Nisa, Jocelyn J. Bélanger, Birga M. Schumpe, Daiane G. Faller

**Affiliations:** New York University Abu Dhabi, Abu Dhabi, UAE

**Keywords:** Environmental social sciences, Psychology and behaviour

**Replying to** Paul C. Stern. *Nature Communications* 10.1038/s41467-020-14653-x (2020)

The essence of this debate boils down to the distinction between intervention and implementation science. On the one hand, intervention science is focused on identifying the most promising interventions for which there is strong research demonstrating effectiveness^1^. The goal is to estimate average main effects, which represent expected impacts averaging out all contextual variables^2^. Evidence synthesis and meta-analysis are key elements of intervention science. In particular, meta-analyses of randomised controlled trials are at the top of the hierarchy of evidence produced in intervention science^3^.

On the other hand, implementation science is focused on the translation of intervention science into practice^4^. Implementation science seeks to close the gap between what we know and what we do, by identifying and addressing the factors that may moderate the effectiveness of interventions in real-world settings^5^. It emphasises on the complexities of the systems (human, social, economic and technological), in which interventions are implemented because interventions that are poorly implemented may not produce the expected benefits^6^.

Due to their distinct focus, intervention and dissemination science tend to use different methodological approaches. Intervention science is mainly focused on establishing causal effects, particularly in the field, whereas implementation science is mainly focused on capturing contextual nuances with multi-method, often non-experimental research designs^7^.

What Stern is ultimately saying is that implementation science is crucial to advance the knowledge about how to best mitigate climate change in households. We say—agreed but—that is not our focus. Our work is in intervention science. This distinction motivated our paper^8^, as well as our response to the arguments presented by Stern, which we cover in turn.

First, Stern attributes the small estimates we report to the fact that our analysis focuses on behavioural interventions acting alone, and he argues that the combination of behavioural interventions with other types of interventions is more effective. We would like to clarify that our results do not imply that all behavioural interventions have little or no effect. Our results show that average effects are small, but that some interventions (social comparison and nudges) are more effective than others. Stern seems to claim that only selected best-case estimates^9^ should be taken as an indication of how effective behavioural interventions are. We respectfully disagree. Average effect sizes represent expected impacts^1,2^, and these average effects are a fundamental benchmark for intervention and policy making^3^. Estimating average main effects from field experiments and large sample sizes is an appropriate method to draw general conclusions because it holds all other factors constant^1^. We agree with Stern that the main effects of behavioural interventions may be moderated by contextual factors, and that bundles of interventions may be more effective when including behavioural and non-behavioural strategies (as we state in page 1; page 2 paragraph 6; page 8 paragraph 2; page 10 paragraph 3). However, these interaction effects need to be carefully tested. Yet, most of the long-standing literature Stern alludes to about combined interventions (e.g., refs. ^9–12^) did not test interaction effects in a rigorous way (i.e., with experimental factorial designs), thus not conclusively estimating the marginal contribution of combined versus behavioural interventions acting alone. It may well be the case that bundles of interventions are more effective, but this needs to be corroborated by causal evidence, and it does not diminish the relevance of isolating the average impact of behavioural interventions.

Second, Stern proposes that the small effects we report could also be due to our focus on frequent versus single-mitigation actions, claiming that the latter is both more relevant to reduce carbon emissions and more difficult to test in field experiments. Although the debate about technical impact was beyond our research goals (our page 10 paragraph 5), we would like to clarify that it is not consensually accepted that single actions are more consequential to mitigate climate change than frequent actions. There are estimates^13^ that eating fewer animal products (a daily occurring behaviour) would reduce an individual’s footprint by 22%, whereas the purchase of a fuel-efficient vehicle (a one-time action) would reduce an individual’s footprint by 9%. We also remind that our results show (pages 3–5) that the impact of behavioural interventions to promote the purchase of energy-efficient appliances (a one-time action) is very small and only marginally significant (*d* = −0.036, 95% CI: −0.129 to 0.058). Moreover, we cautioned the reader (our page 10 paragraph 4) that our analysis did not include some potentially important household actions due to lack of causal field evidence. This is a research caveat, not a serious challenge to our conclusions. Lastly, we also consider a flawed assumption, the notion that single actions are more difficult to experimentally test than frequent actions*.* All field experiments implementing interventions in naturalistic settings are difficult, time-consuming projects, and logistically complex. But testing single actions in the field, such as the purchase of fuel-efficient vehicles^14^, is not uniquely more difficult than testing frequent habitual actions (e.g., daily household energy consumption), which require longer and more intrusive data collection procedures^15^. Therefore, given that we included all available causal field evidence (up to 2018), it is unclear on which grounds Stern is making the claim that because this paper barely addresses actions with high potential impact, the practical relevance of the meta-analysis is open to question. This claim is based on unverified assumptions or evidence of lower methodological quality.

Third, Stern misinterprets our estimates of effect size as the single best estimate of the amount of change that can be achieved. We do not claim to identify the single best effects that can be achieved by behavioural interventions, but the average estimates of the impact that has been achieved to date (our page 4 paragraph 1; page 11 paragraphs 4–7). This means that we estimated the average impacts that can be expected when using a particular type of intervention (e.g., information, appeals and nudges). The discussion about interaction effects also presumes an average—not the best—expected impact from some combination of stimuli. Stern also appears to hold the view that changing mitigation behaviours in households is too complex and multilayered to render an experimental analysis meaningful. We respectfully disagree. We understand Stern’s point, and we do not dismiss the potential relevance of context. However, Stern seems to place a greater emphasis on contextual variables rather than on the effectiveness of the intervention per se. It is unclear which grounds exist for such argument. Some studies suggest that behaviour may change depending on context^11,12^, but this does not necessarily mean that the context is more relevant than the main effect of the intervention, and the plasticity of the behaviour under analysis.

In conclusion, our paper provides a roadmap for intervention by estimating average expected effects, and suggesting to policy makers the most promising avenues for action. This does not imply that contextual adjustments are not necessary to achieve or boost effectiveness. Through this combined lens (intervention + implementation science) can we generate practical policy-level implications, and advance research on promoting household action on climate change.

## Data Availability

This work involves no data. Refer to the original article (ref. 8) for access to the original data under discussion.
